# Experimental investigation and constitutive model for lime mudstone

**DOI:** 10.1186/s40064-016-3297-8

**Published:** 2016-09-21

**Authors:** Junbao Wang, Xinrong Liu, Baoyun Zhao, Zhanping Song, Jinxing Lai

**Affiliations:** 1 School of Civil Engineering, Xi’an University of Architecture and Technology, Xi’an, 710055 China; 2 School of Civil Engineering, Chongqing University, Chongqing, 400045 China; 3 Department of Civil Engineering and Architecture, Chongqing University of Science and Technology, Chongqing, 401331 China; 4 School of Highway, Chang’an University, Xi’an, 710064 China

**Keywords:** Lime mudstone, Mechanical property, Test, Constitutive model

## Abstract

In order to investigate the mechanical properties of lime mudstone, conventional triaxial compression tests under different confining pressures (0, 5, 15 and 20 MPa) are performed on lime mudstone samples. The test results show that, from the overall perspective of variation law, the axial peak stress, axial peak strain and elastic modulus of lime mudstone tend to gradually increase with increasing confining pressure. In the range of tested confining pressure, the variations in axial peak stress and elastic modulus with confining pressure can be described with linear functions; while the variation in axial peak strain with confining pressure can be reflected with a power function. To describe the axial stress–strain behavior in failure process of lime mudstone, a new constitutive model is proposed, with the model characteristics analyzed and the parameter determination method put forward. Compared with Wang’ model, only one parameter *n* is added to the new model. The comparison of predicted curves from the model and test data indicates that the new model can preferably simulate the strain softening property of lime mudstone and the axial stress–strain response in rock failure process.

## Background

With the development of economy, a large number of rock projects are emerging. To guarantee the safety of these projects, it is very important to study the mechanical properties of rock (Singh, et al. 2015; Nadimi et al. 2011; Wang et al. 2014; Yang et al. 2015; Nadimi and Shahriar 2014; Chen et al. 2016a, b; Lai et al. 2016), which as a kind of natural geological material contains many flaws (for instance, joints, micro cracks, faults, etc.). The existence of these flaws complicates the mechanical behavior of rock.

 In the past decades, many efforts have been directed toward the study on mechanical properties of rock. Among them, experimental investigation and constitutive relation are two important aspects. Taking experimental study on mechanical properties of rock as an example, Chen et al. (2016a, b) conducted a series of conventional triaxial compression tests on granite to investigate the pre-failure damage and found that the damage increases slowly before the reversal of volumetric strain and accelerates quickly afterwards. Xia and Zhou (2010) studied the failure process of brittle rock under uniaxial and triaxial compression. The results revealed that rock failure is caused by axial splitting under uniaxial compression; as the confining pressure increases, rock failure occurs in a few localized shear planes and the rock mechanical behavior is changed from brittle to ductile. To study the mechanical strength and deformation of sandstone, Zhang et al. (2015a, b) performed a series of mechanical tests on sandstone samples. They showed that there is a clear transition from volumetric compressibility to dilatancy and a strong dependency on confining pressure. Yang et al. (2012) carried out conventional triaxial compression and “reducing confining pressure” experiments on red sandstone. The test results showed that the post-peak axial deformation characteristics of red sandstone change as the confining pressure are increased from 5 to 65 MPa and Young’s modulus of red sandstone increases nonlinearly with increasing confining pressure. Kahraman and Alber (2008) investigated the uniaxial and triaxial strength of a fault breccia. They found that the uniaxial and triaxial strength of such heterogeneous rocks as fault breccia varies with the proportion of blocks in the sample, the relative strength of the blocks and host matrix and the size of the tested specimen. Hu et al. (2014) tested the mechanical and poromechanical behavior of claystone. The results indicated that the pore pressure, under low confining stress, undergoes a transition from increase to decrease due to the evolution of volumetric strain from contraction to dilatation and this transition gradually disappears under high confining stress. In order to investigate deep reservoir rock properties in High Pressure and High Temperature environments, triaxial tests were performed on Carthage marble and Crab Orchard sandstone samples by Zhang et al. (2015a, b). It was found that with the increase of confining pressure, the Carthage marble changes from strain-softening to strain-hardening, and the Crab Orchard sandstone exhibits brittle or strain-softening behavior; at high temperature, tensile cracks and axial splitting are observed on both rocks; strength of the two rock types is directly dependent on confining pressure and inversely related to temperature. Also, the influence of loading path (Yang et al. 2011; Lee et al. 1999), preexisting flaws (Huang et al. 2015; Yu et al. 2015) and stress state factor (Alexeev et al. 2008) on rock mechanical properties are studied.

For the study on constitutive relation of rock, Taheri and Tani (2013) carried out two series of multiple-step loading triaxial compression tests on a sedimentary soft rock of mudstone. Furthermore, they proposed a multiple-step loading damage model to simulate multiple-step loading triaxial compression test results. The first series was to determine the geotechnical parameters to describe the multiple-step loading damage model, and the second series was to verify the model. The results demonstrated that the proposed multiple-step loading damage model is powerful in simulating multiple-step loading triaxial compression tests on the mudstone. Indraratna et al. (2014) developed a multiphase constitutive model using a critical state framework which includes a kinematic yield locus and a modified stress-dilatancy approach. This model is able to quantify the role of fouling on the permanent strains of the fouled ballast. On the basis of a combination of plasticity theory and the theory of damage mechanics along the lines of a damage-plastic model, Unteregger et al. (2015) presented a constitutive model to describe the nonlinear mechanical behavior of different types of intact rock. The model was validated by numerical simulations of laboratory experiments conducted on specimens of marble, granite and sandstone. Asadollahi et al. (2010) modified the Barton’s empirical model for rock fractures in order to address its limitations and validated it by conducting a series of direct shear tests. Siddiquee et al. (2013) developed a phenomenological model for soft rock based on the results of a series of triaxial compression tests conducted on Kobe sandstone with a very high precision measurement. They simulated the plate loading test results successfully using this model. To characterize the mechanical behavior of rock materials under high confining pressures and high strain rates, a dynamic material model was established by Li and Shi (2016). The reliability and accuracy of the model were verified by the simulation of various basic experiments under different loading conditions. The results indicated that this model is capable of capturing the failure of rock materials. To describe rock mechanical behaviors, Deng and Gu (2011), Liu and Zhang (2015), Li et al. (2015a, b) and Wang et al. (2007) constructed statistical damage constitutive models by taking different strength criteria as a random distributed variable of rock mesoscopic element strength, respectively.

In order to better understand the mechanical properties of lime mudstone, conventional triaxial compression tests under different confining pressures are performed on cylindrical lime mudstone samples. Based on the analyses on test results, a new constitutive model is proposed to simulate the axial stress–strain behavior of lime mudstone in this study.

## Experimental investigation

### Materials and test equipment

Lime mudstone samples used in the tests were obtained from the roof stratum of a salt mine in Huai’an, Jiangsu, China. The salt unit is considered as a host rock for underground gas storage. To analyze the stability of the gas storage, it is very important to study the mechanical behavior of the roof stratum. The lime mudstone samples, drilled from depths ranging between 1600 and 1700 m, were processed into cylinders with a diameter of 50 mm and a height of 100 mm, according to the International Society for Rock Mechanics (Fairhurst and Hudson 1999). The average bulk density of the samples is about 2.56 g/cm^3^, and the colors of them are grey and dark grey.

All tests were carried out in an MTS 815 rock material testing system. This machine is mainly used to test the mechanical properties of solid material under complex stress state. It is suitable for tension, uniaxial compression, triaxial compression, creep, relaxation, and cyclic loading tests for rocks. The maximum axial load is 2800 kN, and the maximum confining pressure is 80 MPa.

### Test procedure

To obtain the stress–strain curves of lime mudstone under different confining pressures, four samples (#1, #2, #3 and #4) were chosen to perform the conventional triaxial compression tests under different confining pressures (*σ*_3_) of 0 (#1), 5 (#2), 15 (#3) and 20 MPa (#4). The following four steps are contained in the test procedure. First, the sample to be tested was covered with a rubber sleeve to make it oil-proof. Second, the covered sample was installed in triaxial pressure cell. Third, the confining pressure was applied at a constant rate of 0.1 MPa/s till it reached the desired value. Finally, the axial stress was applied at a constant axial displacement rate of 0.1 mm/min until rock failure.

## Results and analyses

### Deformation behavior

**Fig. 1:**
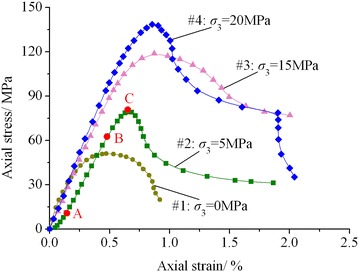
Axial stress–strain curves of lime mudstone under different confining pressures

Figure 1 shows the axial stress–strain curves of the lime mudstone samples under different confining pressures. It can be seen from Fig. 1 that all of the four samples show their strain softening behaviors under different confining pressures, and the rock strength constantly increases with increasing confining pressure.

The deformation behavior of lime mudstone is elaborated with sample #2 (*σ*_3_ = 5 MPa). According to Fig. 1, the axial stress–strain curve of this sample can be divided into four stages (Huang et al. 2015; Liang et al. 2011). (a) Stage I: closure of voids and cracks (before point A). The action of the confining pressure and axial stress gradually closes the open micro structural planes and the micro cracks intrinsically inside the lime mudstone sample and the sample is compacted, which hardens and improves the mechanical properties of rock. At the macro level, the axial stress–strain curve gradually bends to the vertical axis (stress axis) with the increase of strain. (b) Stage II: elastic deformation (the segment between point A and B). At this stage, the deformation of lime mudstone shows linear regular growth with increasing stress, and its axial stress–strain curve is approximate to a straight line. (c) Stage III: plastic deformation (the segment between point B and C). With the further increase of axial stress, the pre-existing cracks inside the lime mudstone sample are gradually developing and new cracks are continuously emerging (Huang et al. 2015; Liang et al. 2011). Therefore, the unrecoverable plastic deformation happens to the sample. At the macro level, the axial stress–strain curve gradually deviates from the straight line and bends to the abscissa axis (strain axis) till it reaches the peak stress (point C). (d) Stage IV: strain softening (after point C). Once the axial stress reaches its peak value, the global internal structures of the lime mudstone sample are damaged and its bearing capacity gradually decreases while its deformation increases continuously.

For the discreteness of the four lime mudstone samples used in the tests, only the axial stress–strain curve of sample #2 (*σ*_3_ = 5 MPa) covers all the aforesaid four stages and that of the other three samples covers only such three stages as the elastic deformation stage, the plastic deformation stage and the strain softening stage, without obvious closure stage of voids and cracks. In addition, sample #1 (*σ*_3_ = 0 MPa) has a relatively short elastic deformation stage but its plastic deformation stage before the peak stress is comparatively long. Moreover, from Fig. 1, it is noted that the axial stress–strain curve of sample #4 (*σ*_3_ = 20 MPa) has an abrupt stress drop. The possible reason is that once the axial stress applied to the sample reaches its peak value, a macroscopic shear fracture plane will be formed and the complete sample is split into two blocks by the shear fracture plane. Then the damaged sample still has a certain bearing capacity because of the existence of friction force between the two blocks. However, the bearing capacity of the damaged sample decreases gradually with increasing strain. When the strain increases to a certain value, further rock failure happens to one of the two blocks, which results in the abrupt stress drop.

### Influence of confining pressure on axial peak stress

**Fig. 2:**
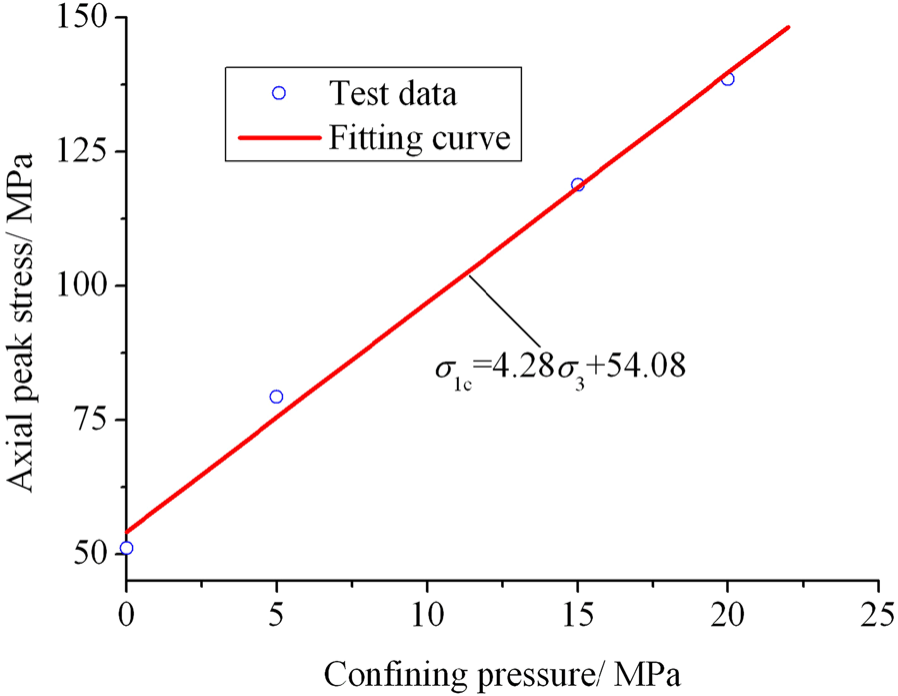
Variation in axial peak stress with confining pressure

The stress corresponding to the peak point of stress–strain curve is the axial peak stress (*σ*_1c_) of rock. Figure 2 shows the variation in axial peak stresses of the four lime mudstone samples with confining pressure. From Fig. 2, it can be seen that the axial peak stress of lime mudstone gradually increases with increasing confining pressure. Many efforts have been devoted to the relationship between rock axial peak stress and confining pressure, with a number of strength criteria proposed. Among them, the linear Mohr–Coulomb strength criterion is extensively used in practical projects (Zhang et al. 2015a, b; Yang et al. 2011; Li et al. 2015a, b) because it has a simple expression and can reflect the essence of shear failure for materials. The linear Mohr–Coulomb strength criterion can be expressed as

1 $$\sigma_{{1{\text{c}}}} = k\sigma_{3} + \sigma_{0}$$

where *σ*_1c_ is the axial peak stress, *σ*_3_ is the confining pressure, *σ*_0_ is usually regarded as the uniaxial compression strength of rock sample, and *k* is a material constant. *σ*_0_ and *k* are related to the cohesion *c* and the internal friction angle *φ* of rock material, which can be expressed in the following forms, respectively.

2 $$k = \frac{1 + \sin \varphi }{1 - \sin \varphi }$$

3 $$\sigma_{0} = \frac{2c\cos \varphi }{1 - \sin \varphi }$$

Fitting analysis is made to the relationship between axial peak stress and confining pressure shown in Fig. 2 using Eq. (1). It can be obtained that

4 $$\sigma_{{1{\text{c}}}} = 4.28\sigma_{3} + 54.08$$

The correlation coefficient *R* = 0.99. Figure 2 also shows the comparison of fitting curve and test data. It can be seen that the fitting curve is in good agreement with the test data, which indicates that the relationship between axial peak stress of lime mudstone and confining pressure conforms well with the linear Mohr–Coulomb strength criterion in the range of 0 MPa ≤ *σ*_3_ ≤ 20 MPa. Then substituting *k* = 4.28 and *σ*_0_ = 54.08 MPa into Eqs. (2) and (3), the cohesion and internal friction angle of lime mudstone can be determined as *c* = 13.07 MPa and *φ* = 38.41°, respectively.

Here it should be noticed that the uniaxial compressive strength (*σ*_3_ = 0 MPa) of lime mudstone calculated with Eq. (4) is 54.08 MPa, while its actual uniaxial compressive strength is 51.14 MPa, lower than the calculated value. That is because the Mohr–Coulomb strength criterion reflects the essence of material shear failure, but the tensile strength of rock is relatively low and in the circumstance of uniaxial compression, the horizontal tensile stress produced by axial compression inside the sample tends to reach the tensile strength first and thus causes the horizontal tensile failure of rock, not shear failure. Therefore, the actual uniaxial compressive strength of rock is generally lower than that calculated with the Mohr–Coulomb formula.

### Influence of confining pressure on axial peak strain

**Fig. 3:**
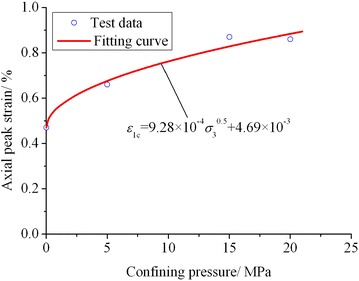
Variation in axial peak strain with confining pressure

The strain corresponding to the peak point of stress–strain curve is the axial peak strain (*ε*_1c_) of rock. According to the conventional triaxial compression test results of marble (Yang et al. 2005) and coal (Yang et al. 2006) under different confining pressures, the axial peak strains of marble and coal show linear increase with increasing confining pressure. Our test data indicate that as for the lime mudstone samples #1, #2 and #3, their axial peak strains constantly increase with the increase of confining pressure, while the axial peak strain of sample #4 is basically identical with that of sample #3. This is mainly due to the discreteness and heterogeneity of the sample. From the overall perspective of variation law, the axial peak strain of lime mudstone tends to gradually increase with increasing confining pressure. Through fitting analysis, the variation in axial peak strain of lime mudstone with confining pressure may be approximately described with the power function expressed by Eq. (5) and the correlation coefficient *R* = 0.98. The comparison of fitting curve and test data is presented in Fig. 3. It can be seen that the fitting curve is in good agreement with the test data.

5 $$\varepsilon_{{1{\text{c}}}} = 9.28 \times 10^{ - 4} \sqrt {\sigma_{3} } + 4.69 \times 10^{ - 3}$$

where *ε*_1c_ is the axial peak strain.

### Influence of confining pressure on elastic modulus

For different types of rocks, the confining pressure has different influence on their elastic modulus. You (2003) pointed out that the elastic modulus of the macroscopically homogeneous and compacted rock sample or that only with local flaws is independent of confining pressure. However, for the sample with joints and cracks, the increased confining pressure may close the joints, cracks, etc., and the friction force between the fissure surfaces may be increased so as to improve the rock elastic modulus.

**Fig. 4:**
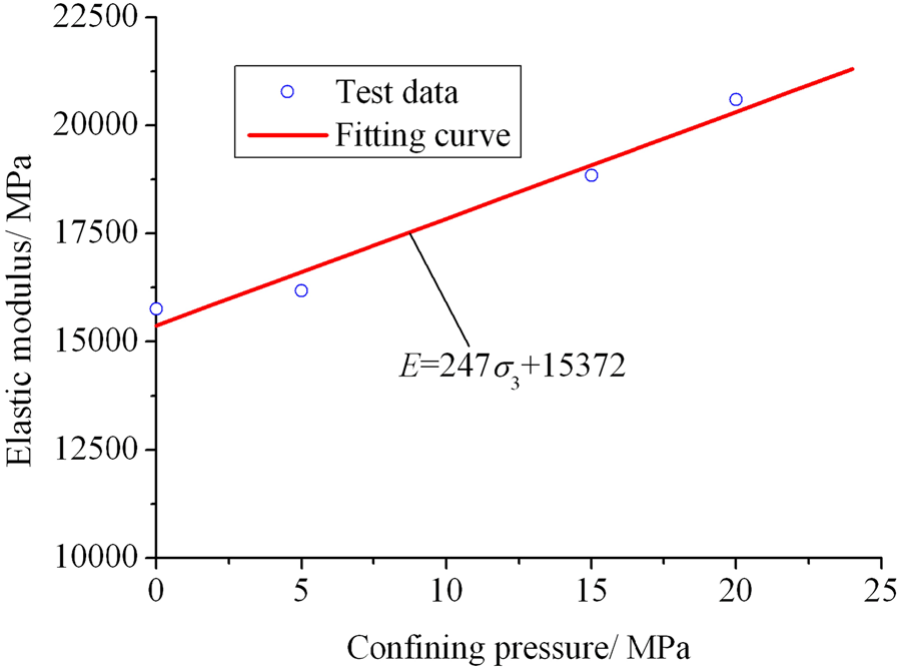
Variation in elastic modulus with confining pressure

Here, the elastic modulus of rock is defined as the slope of the axial stress–strain curve at elastic deformation stage (At this stage, the axial stress–strain curve is approximate to a straight line, and its slope remains basically invariable). Then, according to the axial stress–strain curves shown in Fig. 1, the elastic modulus of lime mudstone samples can be determined as 15,751 (#1), 16,179 (#2), 18,833 (#3) and 20,589 MPa (#4), respectively. Figure 4 illustrates the variation in elastic modulus of the four lime mudstone samples with confining pressure. As shown in Fig. 4, the elastic modulus of lime mudstone gradually increases with the increase of confining pressure, and its variation law may be well fitted with the linear function expressed by Eq. (6) and the correlation coefficient *R* = 0.98.

6 $$E = 247\sigma_{3} + 15372$$

where *E* is the elastic modulus.

### Influence of confining pressure on failure mode

**Fig. 5:**
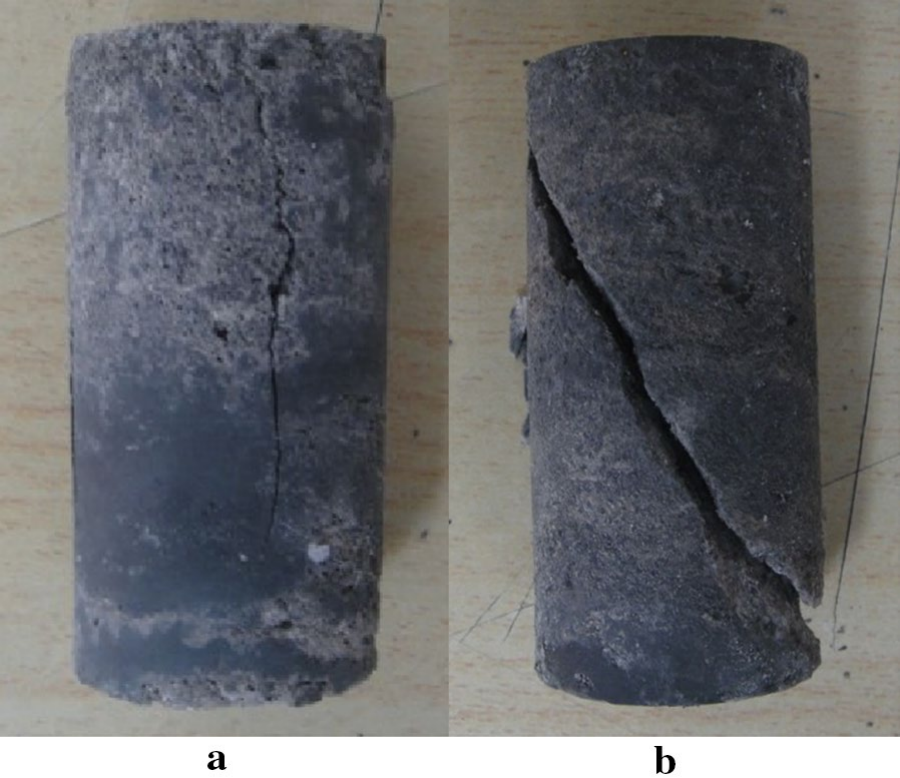
Failure modes of lime mudstone. **a** Uniaxial failure. **b** Triaxial failure

The failure modes of lime mudstone samples under uniaxial (*σ*_3_ = 0 MPa) and triaxial (*σ*_3_ > 0 MPa) compression are presented in Fig. 5. It is easy to see that the failure modes of lime mudstone are different under uniaxial and triaxial compression. Under uniaxial compression, the lime mudstone takes on a horizontal tensile splitting failure mode, during which the tensile splitting crack parallel to the axis appears. The reason is that the tensile strength of rock is far lower than its shear strength and the horizontal tensile stress inside the sample produced by the axial compression reaches its tensile strength first. This is different from the failure mode of coarse marble under the condition of uniaxial compression (Yang et al. 2011). Under triaxial compression, the lime mudstone shows a typical shear failure mode, and the shear plane at a certain angle with the horizontal plane is observed. That is because the action of confining pressure is equivalent to improving the horizontal tensile strength of the rock sample and can restrain the appearance and development of the horizontal tensile cracks.

## Constitutive model

### Proposal of the model and determination method of parameters

Duncan and Chang (1970) hyperbola model is widely used in geotechnical engineering because it has a simple expression and is able to reflect the non-linear property of geotechnical materials. However, this model can only simulate the strain hardening behavior of materials. In order to describe the strain softening property of oil sand, Wang et al. (2015) proposed an improved hyperbola model, with its axial stress–strain relation given by

7 $$\sigma_{1} = \frac{{\varepsilon_{1} \left( {A\varepsilon_{1} + B} \right)}}{{\varepsilon_{1}^{2} + C}}$$

where *σ*_1_ is the axial stress, *ε*_1_ is the axial strain, and *A*, *B* and *C* are material parameters.

The aforesaid model is able to preferably reflect the strain softening property of homogeneous materials, such as oil sand. However, when this model is used to simulate the failure process of lime mudstone, it is found that it has no ideal fitting results in the failure stage (strain softening stage) after the peak point. To precisely simulate the stress–strain response of lime mudstone, a new model with three parameters is proposed. The axial stress–strain relation of this new model is given by

8 $$\sigma_{1} = \frac{{a\varepsilon_{1} + \varepsilon_{1}^{n} }}{{1 + b\varepsilon_{1}^{n} }}$$

where *a*, *b* and *n* are material parameters and *n* ≥ 1.

The characteristics of the model and the parameter determination method are elaborated as follows.

According to Eq. (8), when *ε*_1_ → ∞, the residual strength of rock *σ*_∞_ = 1/*b*, which indicates that 1/*b* represents the residual strength of rock.

Taking the derivative with respect to axial strain *ε*_1_ in Eq. (8), the expression of the tangent elastic modulus *E*_t_ may be obtained to be

9 $$E_{\text{t}} = \frac{{{\text{d}}\sigma_{1} }}{{{\text{d}}\varepsilon_{1} }} = \frac{{a + n\varepsilon_{1}^{n - 1} + \left( {1 - n} \right)ab\varepsilon_{1}^{n} }}{{\left( {1 + b\varepsilon_{1}^{n} } \right)^{2} }}$$

It can be seen from Eq. (9) that the initial tangent elastic modulus *E*_0_ = *a* when *ε*_1_ = 0. This indicates that parameter *a* represents the initial tangent elastic modulus of rock. To get better fitting results, parameter *a* may be replaced with the rock elastic modulus *E* (the slope of the axial stress–strain curve at the elastic deformation stage).

It is assumed that the axial peak stress of rock is *σ*_1c_, the corresponding axial peak strain is *ε*_1c_. Since the tangent elastic modulus at the peak point of the rock axial stress–strain curve is 0, i.e., *E*_t_ = 0, according to Eq. (9), it can be obtained

10 $$a + n\varepsilon_{{1{\text{c}}}}^{n - 1} + \left( {1 - n} \right)ab\varepsilon_{{1{\text{c}}}}^{n} = 0$$

Based on Eqs. (10), (11) can be obtained.

11 $$b = \frac{1}{{\left( {n - 1} \right)\varepsilon_{{1{\text{c}}}}^{n} }} + \frac{n}{{a\left( {n - 1} \right)\varepsilon_{{1{\text{c}}}} }}$$

Assuming that the axial stress–strain relation of rock sample may be described with Eq. (8), it can be expressed at the peak point as

12 $$\sigma_{{1{\text{c}}}} = \frac{{a\varepsilon_{{1{\text{c}}}} + \varepsilon_{{1{\text{c}}}}^{n} }}{{1 + b\varepsilon_{{1{\text{c}}}}^{n} }}$$

Equation (12) can be rewritten as

13 $$a\varepsilon_{{1{\text{c}}}} - \sigma_{{1{\text{c}}}} = \left( {b\sigma_{{1{\text{c}}}} - 1} \right)\varepsilon_{{1{\text{c}}}}^{n}$$

Substituting Eq. (11) into Eq. (13), we get

14 $$a\varepsilon_{{1{\text{c}}}} + \varepsilon_{{1{\text{c}}}}^{n} = \frac{n}{n - 1}\sigma_{{1{\text{c}}}} \left( {1 + \frac{{\varepsilon_{{1{\text{c}}}}^{n - 1} }}{a}} \right)$$

According to Eqs. (11) and (14), as long as the axial peak stress *σ*_1c_, axial peak strain *ε*_1c_ and elastic modulus *a* (*E*) under a certain confining pressure are given, the model parameters *n* and *b* can be determined under this confining pressure. From the analyses on the test results in “Results and analyses” section, it is found that *σ*_1c_, *ε*_1c_ and *a* (*E*) are all dependent on confining pressure *σ*_3_, thus parameters *n* and *b* are inevitably dependent on *σ*_3_. To predict the axial stress–strain behavior of lime mudstone under an arbitrary confining pressure, *σ*_1c_, *ε*_1c_ and *a* (*E*) in Eqs. (11) and (14) may be estimated separately with Eqs. (4)–(6). Then substituting Eqs. (4)–(6) into Eq. (14), we have

15 $$\left( {247\sigma_{3} + 15372} \right)\left( {9.28 \times 10^{ - 4} \sqrt {\sigma_{3} } + 4.69 \times 10^{ - 3} } \right) + \left( {9.28 \times 10^{ - 4} \sqrt {\sigma_{3} } + 4.69 \times 10^{ - 3} } \right)^{n} = \frac{n}{n - 1}\left( {4.28\sigma_{3} + 54.08} \right)\left[ {1 + \frac{{\left( {9.28 \times 10^{ - 4} \sqrt {\sigma_{3} } + 4.69 \times 10^{ - 3} } \right)^{n - 1} }}{{247\sigma_{3} + 15372}}} \right]$$

Similarly, substituting Eqs. (5) and (6) into Eq. (11), we can obtain

16 $$b = \frac{1}{{\left( {n - 1} \right)\left( {9.28 \times 10^{ - 4} \sqrt {\sigma_{3} } + 4.69 \times 10^{ - 3} } \right)^{n} }} + \frac{n}{{\left( {247\sigma_{3} + 15372} \right)\left( {n - 1} \right)\left( {9.28 \times 10^{ - 4} \sqrt {\sigma_{3} } + 4.69 \times 10^{ - 3} } \right)}}$$

Equations (15) and (16) indicate that parameters *n* and *b* are relevant to confining pressure *σ*_3_. For a given confining pressure, parameter *n* corresponding to this confining pressure may be determined by solving Eq. (15). It should be noticed that Eq. (15) cannot be written to be an explicit form, so it is difficult to solve it directly and its solution (*n*) may be obtained with iterative method using mathematical software. Then substituting the value of *n* into Eq. (16), parameter *b* corresponding to this confining pressure can be determined. Meanwhile, parameter *a* (*E*) corresponding to this confining pressure may be estimated with Eq. (6). Thus, according to Eqs. (6), (15) and (16), the model parameters *a* (*E*), *b* and *n* under an arbitrary confining pressure can be determined. Then the axial stress–strain behavior of lime mudstone under an arbitrary confining pressure can be predicted. This determination method for parameters is conducive to predicting the axial stress–strain response of rock.

### Influence of parameter *n* on the model

**Fig. 6:**
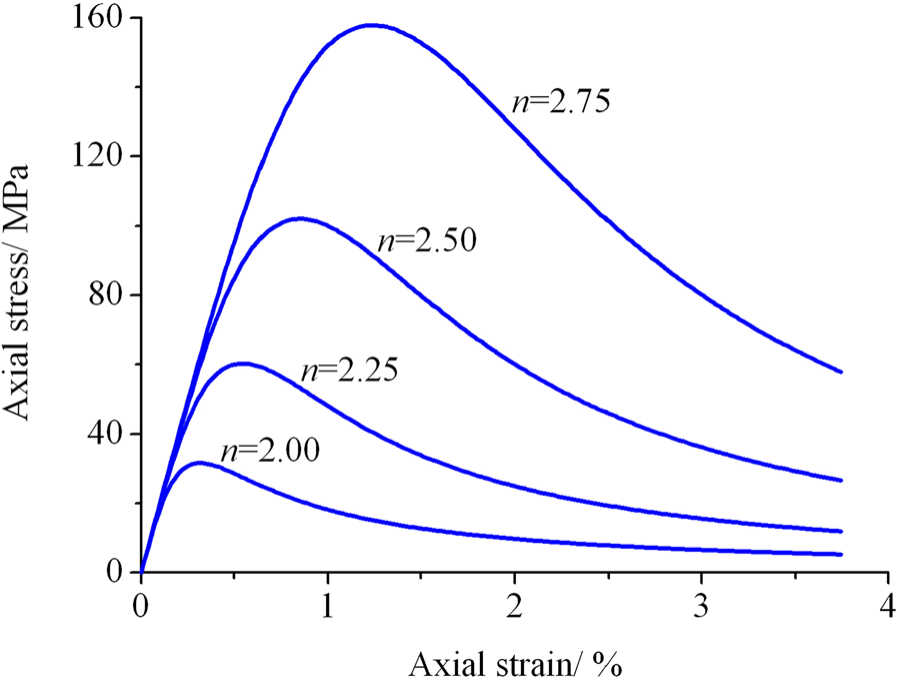
Influence of parameter *n* on axial stress–strain curve

According to the analyses in “Proposal of the model and determination method of parameters” section, parameter *a* represents the rock elastic modulus, the reciprocal of parameter *b* represents the residual strength, and parameter *n* is a fitting parameter, the main function of which is to change the shape of the rock stress–strain curve so as to better fit the test results. In Eq. (8), let *a* = 2 × 10^4^ MPa and *b* = 1 × 10^5^ MPa^−1^. Figure 6 illustrates the influence of parameter *n* on the model axial stress–strain curve.

From Fig. 6, it can be observed that the elastic deformation stages of the axial stress–strain curves corresponding to different values of parameter *n* coincide with each other. As the values of parameter *n* increase, both the axial peak stress and axial peak strain are constantly increasing; meanwhile, the strain softening property of material is more and more obvious. The model proposed in this paper may degrade into Duncan–Chang model under the condition of *n* = 1. Then only the strain hardening property of material can be described and its strain softening behavior cannot be reflected by this model. For different rock samples, the shape of the axial stress–strain curve can be changed by adjusting the value of parameter *n* so as to better fit the test results. Therefore, the proposed model in this paper has better adaptability in describing the axial stress–strain response of rock material.

## Validation of the model

**Fig. 7:**
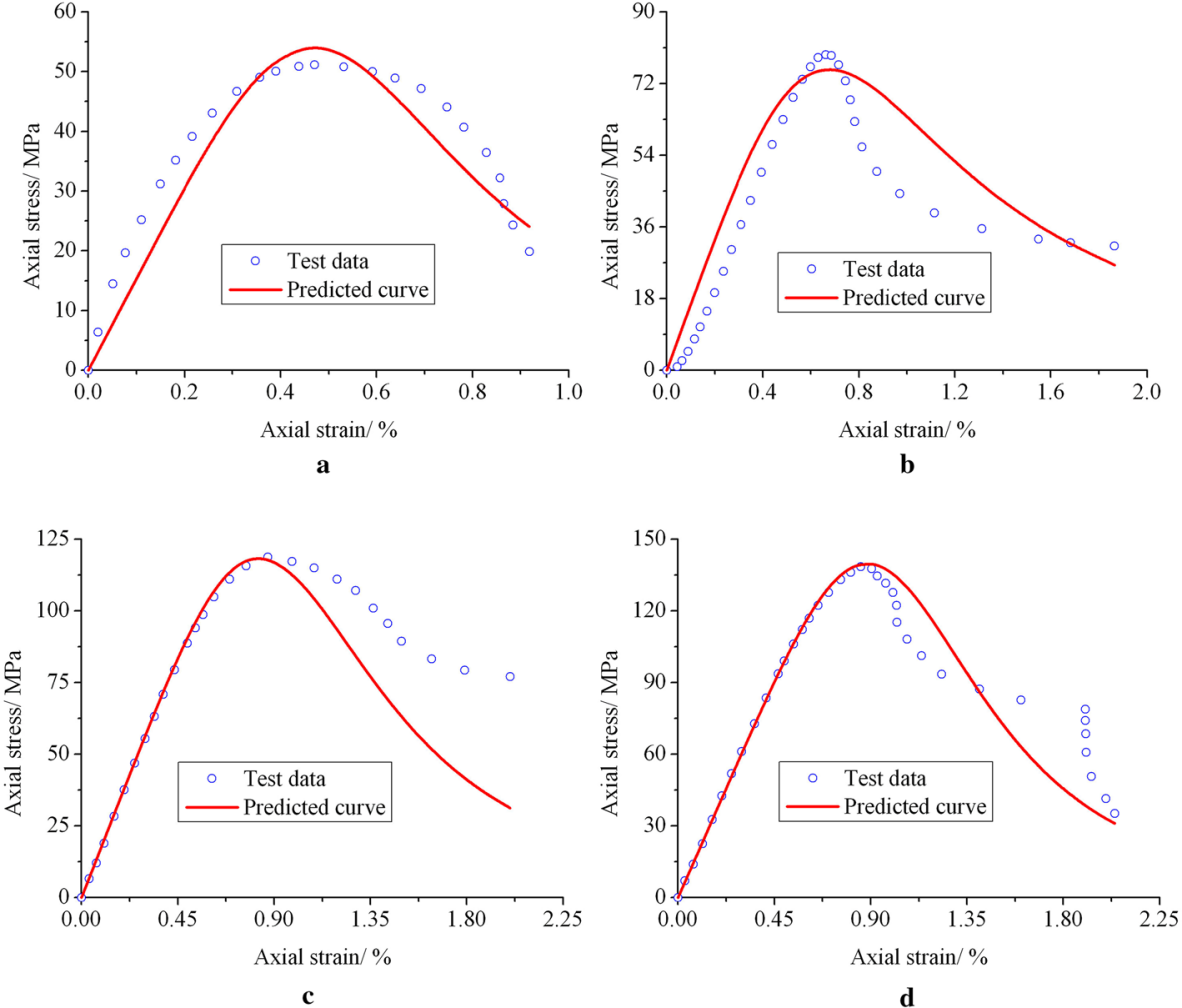
Comparison of predicted curves and test data. **a** *σ*_3_ = 0 MPa. **b** *σ*_3_ = 5 MPa. **c** *σ*_3_ = 15 MPa. **d** *σ*_3_ = 20 MPa

The conventional triaxial compression test results of lime mudstone shown in Fig. 1 are utilized to evaluate the rationality of the model proposed in this paper. First, the model parameters under such four different confining pressures as *σ*_3_ = 0 MPa, *σ*_3_ = 5 MPa, *σ*_3_ = 15 MPa and *σ*_3_ = 20 MPa are determined according to Eqs. (6), (15) and (16). For instance, when *σ*_3_ = 0 MPa, parameter *a* can be determined as *a* = 15372 MPa by substituting *σ*_3_ = 0 MPa into Eq. (6); and parameter *n* can be determined as *n* = 3.99 by substituting *σ*_3_ = 0 MPa into Eq. (15). Substituting *σ*_3_ = 0 MPa and *n* = 3.99 into Eq. (16), parameter *b* can be determined as *b* = 6.60 × 10^8^ MPa^−1^. Then substituting *a* = 15372 MPa, *b* = 6.60 × 10^8^ MPa^−1^ and *n* = 3.99 into Eq. (8), the axial stress–strain equation of lime mudstone under *σ*_3_ = 0 MPa can be obtained and the predicted axial stress–strain curve can be further drawn. Under other confining pressures, the parameter determination method and procedure are similar. *σ*_3_ = 5 MPa: *a* = 16607 MPa, *b* = 1.98 × 10^6^ MPa^−1^ and *n* = 3.05; *σ*_3_ = 15 MPa: *a* = 19077 MPa, *b* = 6.39 × 10^7^ MPa^−1^ and *n* = 3.98; *σ*_3_ = 20 MPa: *a* = 20312 MPa, *b* = 5.13 × 10^8^ MPa^−1^ and *n* = 4.51. Figure 7 presents the comparison of predicted curves and test data.

It can be seen from Fig. 7 that, for the complexity of rock mechanical properties, some errors are found to exist between predicted curves and test results. Figure 7a shows that the tested axial stress–strain curve of sample #1 (*σ*_3_ = 0 MPa) has a shorter elastic deformation stage but a longer plastic deformation stage before the peak point, and the predicted curve from the model is relatively in poor agreement with the test data before the peak point. Figure 7b indicates that the tested axial stress–strain curve of sample #2 (*σ*_3_ = 5 MPa) contains closure stage for voids and cracks, while the mechanical properties of rock in this stage are not considered in the model, so the predicted curve from the model is also relatively in poor agreement with the test data before the peak point. However, the comparison of predicted curves from the model and test data illustrates that, from the overall perspective, the new model proposed in this paper can preferably simulate the strain softening property of lime mudstone and the axial stress–strain response in rock failure process.

## Conclusions

The conventional triaxial compression test results under different confining pressures (0, 5, 15 and 20 MPa) show that, in the range of 0 MPa ≤ *σ*_3_ ≤ 20 MPa, the variations in axial peak stress and elastic modulus of lime mudstone with confining pressure can be described with linear functions; while the variation in axial peak strain of lime mudstone with confining pressure can be reflected with a power function. Moreover, the lime mudstone exhibits a horizontal tensile splitting failure mode under uniaxial compression and a typical shear failure mode under triaxial compression.

In order to precisely describe the axial stress–strain behavior in failure process of lime mudstone, a new constitutive model is proposed, with the model characteristics analyzed and the parameter determination method put forward. Then the rationality of the model is validated using the conventional triaxial compression test results of lime mudstone. The model parameters under different confining pressures are determined first, and then the predicted axial stress–strain curves are obtained by substituting the corresponding parameters into the constitutive equation. The comparison of predicted curves from the model and test data indicates that the new model can preferably simulate the strain softening property of lime mudstone and the axial stress–strain response in rock failure process.

## Abbreviations

*σ*_3_: confining pressure
*σ*_1c_: axial peak stress
*σ*_0_: uniaxial compression strength of rock material
*k*: material constant
*c*: cohesion of rock material
*φ*: internal friction angle of rock material
*ε*_1c_: axial peak strain
*E*: elastic modulus
*σ*_1_: axial stress
*ε*_1_: axial strain
*A*: material parameter of Wang’ model
*B*: material parameter of Wang’ model
*C*: material parameter of Wang’ model
*a*: material parameter of the model proposed in this paper
*b*: material parameter of the model proposed in this paper
*n*: material parameter of the model proposed in this paper
*σ*_∞_: residual strength
*E*_t_: tangent elastic modulus
*E*_0_: initial tangent elastic modulus
